# Chronic pain has a strong impact on quality of life in facioscapulohumeral muscular dystrophy

**DOI:** 10.1002/mus.25991

**Published:** 2017-11-07

**Authors:** Germán Morís, Libby Wood, Roberto FernáNdez‐Torrón, José Andrés González Coraspe, Chris Turner, David Hilton‐Jones, Fiona Norwood, Tracey Willis, Matt Parton, Mark Rogers, Simon Hammans, Mark Roberts, Elizabeth Househam, Maggie Williams, Hanns Lochmüller, Teresinha Evangelista

**Affiliations:** ^1^ MRC Centre for Neuromuscular Diseases, Institute of Genetic Medicine Newcastle University, Central Parkway Newcastle upon Tyne NE1 3 BZ United Kingdom; ^2^ Neuromuscular Disorders Unit Neurology Department, Hospital Donostia San Sebastián Spain; ^3^ Neuroscience Area Biodonostia Health Research Institute San Sebastián Spain; ^4^ Center for Biomedical Research in the Neurodegenerative Diseases (CIBERNED) Network Instituto Carlos III, Ministry of Economy and Competitiviness Madrid Spain; ^5^ UCL MRC Centre for Neuromuscular Diseases, Institute of Neurology London United Kingdom; ^6^ Department of Clinical Neurology John Radcliffe Hospital Oxford United Kingdom; ^7^ Department of Neurology King's College Hospital London United Kingdom; ^8^ The Robert Jones and Agnes Hunt Orthopaedic Hospital Oswestry United Kingdom; ^9^ Institute of Medical Genetics University Hospital of Wales Cardiff United Kingdom; ^10^ Wessex Neurological Centre University Hospital of Southampton Southampton United Kingdom; ^11^ Department of Neurology Salford Royal NHS Foundation Trust Salford United Kingdom; ^12^ Department of Neurology Derriford Hospital, Plymouth Hospitals NHS Trust Plymouth United Kingdom; ^13^ Bristol Genetics Laboratory, Southmead Hospital, North Bristol NHS Trust Bristol United Kingdom

**Keywords:** facioscapulohumeral dystrophy, INQoL, pain, patient registry, patient reported outcome measures, quality of life

## Abstract

**Introduction:**

Earlier small case series and clinical observations reported on chronic pain playing an important role in facioscapulohumeral dystrophy (FSHD). The aim of this study was to determine the characteristics and impact of pain on quality of life (QoL) in patients with FSHD.

**Methods:**

We analyzed patient reported outcome measures collected through the U.K. FSHD Patient Registry.

**Results:**

Of 398 patients, 88.6% reported pain at the time of study. The most frequent locations were shoulders and lower back. A total of 203 participants reported chronic pain, 30.4% of them as severe. The overall disease impact on QoL was significantly higher in patients with early onset and long disease duration. Chronic pain had a negative impact on all Individualised Neuromuscular Quality of Life Questionnaire domains and overall disease score.

**Discussion:**

Our study shows that pain in FSHD type 1 (FSHD1) is frequent and strongly impacts on QoL, similar to other chronic, painful disorders. Management of pain should be considered when treating FSHD1 patients. *Muscle Nerve* **57**: 380–387, 2018

**Abbreviations**
FSHD: facioscapulohumeral muscular dystrophy
FSHD1: facioscapulohumeral muscular dystrophy type 1
INQoL: Individualised Neuromuscular Quality of Life Questionnaire
IR: interquartile range
JWMDRC: John Walton Muscular Dystrophy Research Centre
NSAIDs: nonsteroidal anti‐inflammatory drugs
QoL: quality of life
PROM: patient reported outcome measures
SF‐MPQ: Short Form of the McGill Pain Questionnaire
*SMCHD1*: structural maintenance of chromosomes flexible hinge domain containing 1

Facioscapulohumeral muscular dystrophy (FSHD) is the second most common muscle condition in adults, with an overall incidence of 1:20,000.^1^, ^2^ FSHD is an autosomal dominant and genetically heterogeneous disorder; in 95% of patients (FSHD1), it is associated with the loss of part of a D4Z4 repeated sequence in chromosome 4q35. In 5% of patients (FSHD2), mutations in the structural maintenance of chromosomes flexible hinge domain containing 1 (*SMCHD1*) gene are found. The D4Z4 methylation status changes in FSHD and the D4Z4 hypomethylation leads to chromatin relaxation of D4Z4 and expression of *DUX4*.^3^

FSHD symptoms usually start around the second decade and are most commonly characterized by asymmetric weakness affecting the face, shoulder, and arms, followed by the distal lower extremities and pelvic girdle. Not all patients have the complete phenotype of FSHD, and clinical severity varies widely among patients, including great variability of weakness within families. Chronic pain is a significant problem in different neuromuscular conditions,^4^, ^5^ such as limb‐girdle muscular dystrophy 1C, myotonic dystrophies, and FSHD^6^, ^7^; in some cases, pain may even be the first disease manifestation.^8^, ^9^ Reports about pain in FSHD‐phenotype patients predate the development of genetic testing.

Recent studies have suggested that pain may be present in the majority of FSHD patients, ranging from 76% to more than 80% of the FSHD population, with 19% reporting severe pain.^5^, ^10^, ^11^, ^12^, ^13^, ^14^, ^15^, ^16^ In some cases, FSHD patients reported severe, difficult to control, multifocal muscle pain as the most disabling aspect of their condition.^9^ Even so, pain is often undertreated. Some studies have shown that pain in FSHD negatively impacts quality of life (QoL) and increases disease burden.^13^, ^15^ However, the data available is scarce, often not FSHD‐specific and usually clinician‐reported. The studies address small heterogeneous cohorts that include other neuromuscular disorders.^5^, ^10^ Moreover, epidemiology, etiology, pathophysiology, and management of pain have not yet been addressed, nor has the interaction between pain and its impact on QoL.

The aim of the study was to determine the frequency, localization, and intensity of pain in the FSHD1 population registered in the U.K. FSHD registry; and to evaluate the influence of pain, age, sex, disease duration, and ambulatory status on QoL.

## MATERIALS AND METHODS

### Patients and Setting

We have analyzed data obtained from the U.K. FSHD Patient Registry that is curated from the John Walton Muscular Dystrophy Research Centre (JWMDRC) at Newcastle University (https://www.fshd-registry.org/uk/participants/questionnaires/index.en.html). The Registry started in May 2013. A cutoff point for data analysis was established in February 2017. This patient driven registry^17^ is based on the recommendations reported at the 171st European Neuromuscular Centre workshop on the care and management of FSHD.^18^ In addition to these items, several patient reported outcome measures (PROM) on pain and QoL and were added after consultation with the patient community about their own research priorities. The registry has received full ethical (Newcastle and North Tyneside 113/NE/0048, February 2013), management (Newcastle upon Tyne Hospitals Trust R&D 6573, February 2013), and data protection (Caldicott February 2013) approvals.

### Measures

#### Pain

The Short Form of the McGill Pain Questionnaire (SF‐MPQ) was used to report pain. The SF‐MPQ is widely used, well‐validated, and reliable and has previously been used in FSHD1.^19^ The SF‐MPQ consists of 15 descriptors (11 sensory and 4 affective) which are rated on an intensity scale as 0 = none, 1 = mild, 2 = moderate or 3 = severe, with higher scores representing more pain. Sensory (ranging from 0 to 33), affective (ranging from 0 to 12), and total pain scores (ranging from 0 to 45) and Present Pain Intensity Index (ranging from 0 to 5) were analyzed. An FSHD specific PROM, named universal pain assessment tool, developed by the multi‐disciplinary team at JWMDRC was also used. The universal pain assessment tool is in the process of validation and it includes sections on pain intensity, current pain and chronic pain, and in addition questions regarding medication and other nonpharmacological therapies (sections of the universal pain assessment tool used in this study are shown in Supplementary Table S1, which is available online). For the purpose of the study, “current pain” was defined as any pain experienced in the last 7 days and “chronic pain” as any persistent pain experienced for at least 12 weeks within a year in the last 5 years. The universal pain assessment tool clearly provided the time definition of current and chronic pain according to study pain definitions. To analyze pain intensity, the pain was considered to be severe when reported as horrible or excruciating.

#### Individualized Neuromuscular Quality of Life Questionnaire

Patients completed the Individualised Neuromuscular Quality of Life Questionnaire (INQoL), a widely used and well‐validated neuromuscular disease specific measure of QoL.^20^, ^21^ The final score for each section and total INQoL score is presented as a percentage of the maximum detrimental impact where 100 is the greatest impact on QoL.

### Data Analysis

Location and dispersion indexes of noncontinuous variables were used to describe the sample with median and interquartile range (IR). Categorical variables were described as percentages. All statistics discussed here are presented as percentages of the total patient answers and not as percentages of the whole cohort unless otherwise stated. For comparison between groups, Chi‐square tests, Mann‐Whitney *U* test, and Kruskal‐Wallis test were used, as appropriate. Correlations were calculated using nonparametric Spearman's coefficient. To evaluate influence of age, age of onset, disease duration, gender, D4Z4 repeat, SF‐MPQ total score, and chronic pain on INQoL we performed a multivariate linear regression. Differences were considered significant at *P* < 0.05, and all statistical tests were two tailed. All data were analyzed with IBM SPSS Statistics 22.

## RESULTS

### Clinical Data

**Figure 1:**
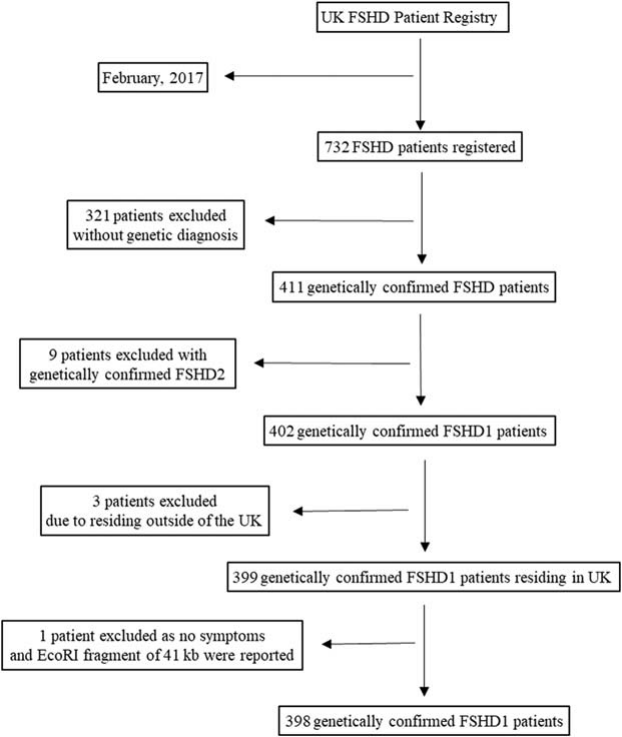
Flow diagram of the progress of participants included in the U.K. FSHD Patient Registry and the number of patients excluded.

Four hundred and two genetically confirmed FSHD1 patients were included in this study. Figure 1 shows the progress of participants included. In total, 398 patients have been included for further analysis. All patients are followed‐up by neurologists with experience in neuromuscular disorders to ensure the FSHD1 diagnosis. Of these 398 UK genetically confirmed FSHD1 patients, 383 (96.2%) answered the SF‐MPQ, 367 (92.2%) and 365 (91.7%) answered current and chronic pain questions in the universal pain assessment tool, respectively, and 340 (85.4%) provided answers to INQoL.

The median age of the entire cohort was 47.0 (interquartile range: 60.6) years. Demographic data is presented in Table 1. In 6 patients, the size of the p13E‐11 *EcoR*I fragments was > 39 kb (3 patients had 39 kb, and 3 patients had 40, 41, and 45 kb, respectively); although, they have neither *SMCHD1* test nor methylation studies. These 6 patients were included in the study because all reported symptoms and the treating neuromuscular specialist confirmed clinical features consistent with FSHD. There was no *Eco*RI fragment information in 45 of the cases.

**Table 1:** Demographic data and frequency of pain

	Total	Males	Females
Epidemiological			
Gender	398	197 (49.6%)	201 (50.4 %)
Age (years)*	47.0 (60.6)	48.0 (25.0)	46.0 (25.0)
Age onset (years)^a^	17.0 (21.2)	19.0 (22.0)^b^	13.5 (22.5)^b^
Disease duration (years)^a^	26.0 (27.0)	24.0 (22.5)	27.5 (30.7)
Genetic test (D4Z4 repeat kb)^a^	25.0 (10.0)	25.0 (10.0)	24.0 (11.3)
Motor function, total (%)			
Ambulatory‐unassisted	193 (49.4%)	91 (47.4%)	102 (51.3%)
Ambulatory‐assisted	145 (37.1%)	78 (40.6%)	67 (33.7%)
Non‐ambulatory	53 (13.6%)	23 (12.0%)	30 (15.1%)
Wheelchair use, total (%)			
No use	244 (62.2%)	125 (65.1%)	119 (59.5%)
Part‐time	94 (24.0%)	44 (22.9%)	50 (25.0%)
Full‐time	54 (13.8%)	23 (12.0%)	31 (15.5%)
SF‐MPQ			
Presence of pain Total (%)	339 (85.2 %)	164 (83.2%)	175 (87.1%)
Sensory Score^a^	5.0 (9.0)	3.0 (9.0)^c^	6.0 (9.0)^c^
Affective Score^a^	1.0 (3.0)	1.0 (3.0)	2.0 (4.0)
Total score^a^	6.0 (12.5)	5.0 (11.5)	7.0 (12.5)
Present Pain Intensity Index^a^	1.0 (2.0)	1.0^a^ (2.0)	1.0 (1.0)
Chronic pain			
Chronic pain Total (%)	203 (55.6%)	92 (50.3%)^c^	111 (61.0%)^c^
Severe chronic pain Total (%)	69 (30.4%)	29 (26.6%)	40 (33.9%)

a Expressed in median and interquartile range.

b P < 0.01.

c P < 0.05.

### Pain

**Figure 2:**
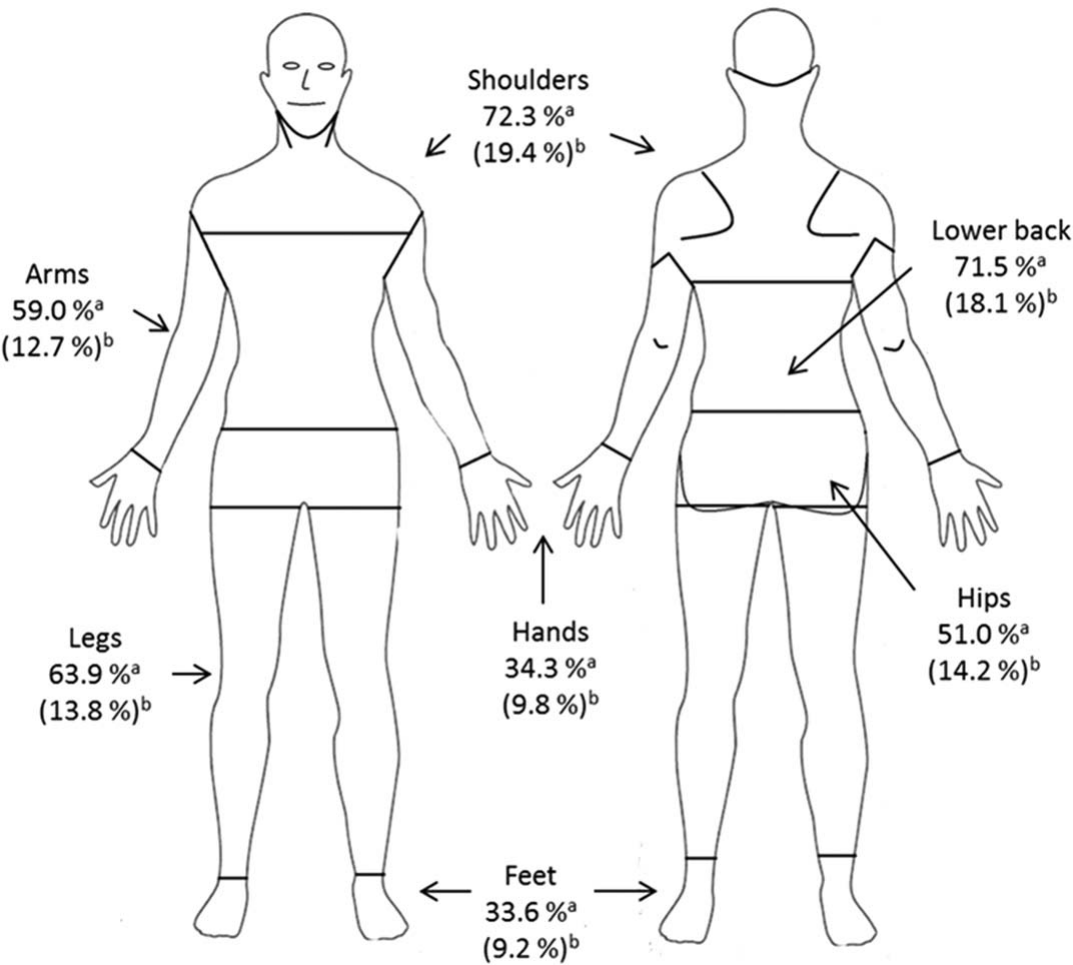
Description of the localization and the severity of the current pain in each localization expressed in percentage. ^a^Percentage of patients suffering current pain in each localization. ^b^Percentage of patients suffering severe current pain (pain rated as horrible or excruciating) in each localization.

Of those completing the SF‐MPQ, 339 patients (88.5%) reported experiencing pain to some degree. The sensory score in females was significantly higher (*P* < 0.05) than in males (Table 1). Three hundred and twenty‐five (88.6%) participants reported experiencing current pain. Figure 2 shows the percentage of patients that reported the highest levels of pain and the pain severity in different locations. The severe lower back pain was reported more frequently in females (33 patients; 17.7%) than in males (14 patients; 11.2%) with statistical significance (*P* < 0.05). No significant differences were observed regarding current pain localization across *EcoR*I fragments size, age of onset or motor function as showed in Table 2. Of the 365 responders, 203 (55.6%) people reported experiencing chronic pain, 69 patients (30.4%) reported this pain as severe. The chronic pain was more frequently reported in females (111 patients; 61.0%) than in males (92 patients, 50.3%) (*P* < 0.05). The most common location of chronic pain was the shoulder joint in 165 patients (45%). No association was seen between chronic pain and *Eco*RI fragments size, age of onset, or motor function.

**Table 2:** Characteristics of pain in different sub‐groups of FSHD1 patients

	Total	SF‐MPQ^a^	Severe current pain localizations Total (%)		Chronic pain Total (%)	
		Total pain score	Shoulder	Lower back	Presence chronic pain	Severe chronic pain
Age of onset (years)						
*0‐9*	107	7.0 (9.0)	13 (17.8%)	19 (24.1 %)	62 (64.6 %)	23 (34.8%)
*10‐19*	104	7.5 (13.0)	14 (20.9%)	11 (16.7%)	53 (54.6%)	19 (33.3%)
*20‐39*	99	6.0 (13.0)	16 (23.5%)	11 (16.7%)	55 (58.5%)	22 (34.4%)
* > 40*	56	5.0 (7.7)	7 (17.1%)	5 (13.5%)	27 (50.0%)	4 (13.3%)
Disease duration (years)						
*0‐19*	138	6.0 (12.0)	22 (22.7%)	13 (15.1%)	75 (56.4%)	22 (26.5%)
*20‐39*	130	9.0 (13.0)	19 (20.2%)	22 (22.9%)	70 (57.4%)	30 (38.5%)
* > 40*	98	12.0 (12.0)	9 (15.5%)	11 (16.7%)	52 (60.5%)	16 (28.6%)
Fragment size, total						
≤ 18 Kb	82	5.0 (14.0)	6 (13.6 %)	14 (25.9 %)	38 (54.3 %)	14 (33.3%)
> 18 Kb	271	6.0 (8.0)	39 (20.5%)	28 (15.5%)	144 (56.9%)	46 (28.9%)
Motor function						
Ambulatory‐unassisted	193	6.0 (10.0)^b^	17 (15.5%)	24 (18.9%)	95 (51.1%)	29 (27.4% )
Ambulatory‐assisted	145	9.0 (13.0)^b^	27 (24.1%)	24 (22.9%)	83 (60.6%)	34 (37.4%)
Non‐ambulatory	53	6.0 (11.0)^b^	3 (8.1%)	3 (9.7%)	28 (58.3%)	6 (20.0%)

a Expressed in median and interquartile range.

b P < 0.05.

### Therapies

**Figure 3:**
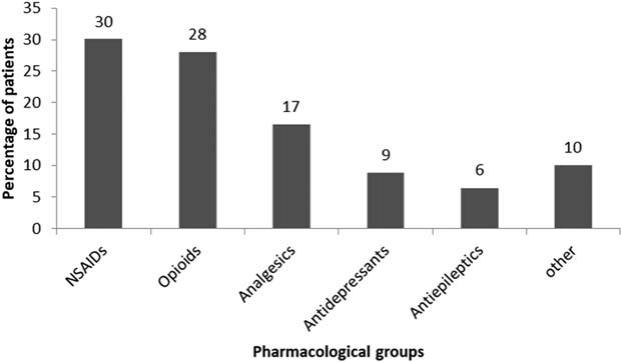
Most frequently pharmacological groups used to manage pain.

**Figure 4:**
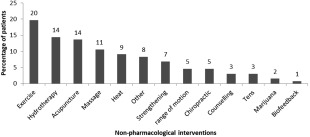
Most frequently nonpharmacologic interventions used to manage pain.

Medication to manage pain was taken by 367 (92.2%) patients. When specified, nonsteroidal anti‐inflammatory drugs (NSAIDs) were the most frequent drugs used followed by opioids as described in Figure 3. Less than half of patients (162 patients, 46.2%) have used physiotherapy to help with pain management; a dramatic reduction in pain was reported by 4.4% (7 patients). Sixty‐two patients (38.8%) reported some reduction in pain, and 80 (50.0%) reported no reduction. Eleven patients (6.9%) experienced an increase in their pain after physiotherapy. We did not find any statistical difference in response to physiotherapy between any groups studied. In this survey, 370 patients (93%) reported using nonpharmacologic therapies to help manage pain (Fig. 4).

### Quality of Life

The median overall INQoL score was 53.1 (IR: 34.3) suggesting relatively moderate sickness‐related dysfunction in this group of FSHD1 patients. The sub‐domains with the highest median scores, indicating the greatest impact on QoL were muscle weakness, body image, and activities. The least impact on QoL is in the areas of muscle locking and relationships. The weakness score in males was significantly higher than in females (*P* < 0.01). No other domain was affected by gender (Table 3). A higher total INQol score is seen in patients with a shorter *Eco*RI fragment (*P* < 0.05). The overall INQol increases with statistical significance with younger age of onset (rho = ‐0.22) and longer disease duration (rho = +0.23) (*P* < 0.05).

**Table 3:** INQoL subscale scores and overall score comparisons between genders, and absence or presence of chronic pain

INQoL		Gender		Chronic pain	
	Total	Males	Females	No	Yes
Weakness score	63.2 (26.4)	73.7 (36.8)^a^	68.4 (47.4)^a^	57.9 (47.4)^a^	79.0 (31.6)^a^
Locking score	10.5 (22.4)	10.5 (10.5)	10.5 (13.2)	10.5 (0.0)^a^	10.5 (26.3)^a^
Pain score	44.7 (31.6)	31.6 (52.6)	36.8 (47.4)	10.5 (15.8)^a^	63.2 (42.2)^a^
Fatigue score	50.0 (34.2)	47.4 (52.6)	47.4 (52.6)	26.3 (42.1)^a^	68.4 (43.4)^a^
Activities score	57.4 (58.6)	52.8 (43.1)	53.2 (54.9)	37.0 (38.5)^a^	67.6 (37.3)^a^
Independence score	50.0 (64.6)	34.7 (53.5)	33.3 (55.6)	25.0 (35.4)^a^	55.6 (50.2)^a^
Relationship score	20.4 (16.7)	20.4 (28.7)	18.5 (35.6)	13.0 (21.3)^a^	30.6 (37.1)^a^
Feelings score	54.2 (56.2)	38.9 (42.4)	41.7 (39.6)	27.8 (33.3)^a^	50.0 (40.3)^a^
Body image score	59.7 (51.4)	55.6 (52.8)	58.3 (55.6)	41.7 (50.0)^a^	61.1 (50.0)^a^
QoL Score	53.1 (34.3)	52.2 (30.0)	51.1 (36.3)	41.1 (30.4)^a^	60.6 (29.5)^a^

Values are expressed in median and interquartile range.

a P < 0.01.

The pain INQoL score is higher in ambulant populations (*P* < 0.01).SF‐MPQ sensory, affective, and total pain scores correlated with INQoL pain score (rho = +0.79, rho = + 0.68, rho = + 0.80, respectively, *P* < 0.01), and INQoL total score (rho = +0.48, rho = + 0.50, rho = + 0.51, respectively, *P* < 0.01). On the other hand, the presence of severe pain in the shoulders, severe lower back pain, chronic pain, and severe chronic pain correlated with the INQoL pain score (rho = +0.54, rho = + 0.46, rho = + 0.67, rho = +0.44, respectively, *P* < 0.01), and the INQoL total score (rho = +0.32, rho = + 0.29, rho = + 0.38, rho = +0.29, respectively, *P* < 0.01). Multiple regression analysis showed that longer disease duration was related to greater deterioration on INQol; interestingly, the total SF‐MPQ score and presence of chronic pain was also directly related to an increase in total INQoL scores as showed in Table 4.

**Table 4:** Association of INQol index with demographic, genetic, and pain presence

	INQoL index	
	Univariate analysis (*P*)	Multivariate analysis (beta, *P*)
Age	0.338	NI
Age of onset	0.001	‐0.057, 0.323
Disease duration	0.001	+ 0.173, 0.003
Gender	0.529	NI
D4Z4 repeat	0.062	NI
SF‐MPQ total score	0.001	+ 0.400, 0.001
Chronic pain	0.001	+0.128, 0.029
		Adjusted R^2^: 0.284

NI, not included in the multivariate analysis since it was non‐significant in univariate analysis.

## DISCUSSION

This study demonstrates that pain is highly prevalent in FSHD1. Our data are consistent with previous studies carried out in smaller mixed disease cohorts, and supports the conclusion that pain is a common complaint in patients with FSHD1, similar to or possibly worse than in other neuromuscular conditions, even though a direct comparative study was not conducted.^5^, ^14^ Furthermore, pain is reported in a higher percentage in our cohort that would be expected in the general population, where is ranges from 10% to 55%.^22^, ^23^ The frequency and intensity of pain reported in our population are similar to what has been reported previously in osteoarthritis or rheumatoid arthritis.^24^, ^25^ Chronic pain is a substantial problem in FSHD1 and needs to be tackled in a holistic way with exercise/physiotherapy, psychosocial intervention and tailored pharmacological treatment.

Gender influences FSHD1 clinical expression. Males are characterized by a lower age at onset of motor impairment and by a more severe disability.^26^ However, our data shows that female FSHD1 patients experience pain more frequently than males, suggesting that clinical impairment is not the only underlying factor leading to pain in FSHD1 patients. Although commonly reported, the mechanisms behind gender differences in pain perception are unknown; biopsychosocial mechanisms, influence of sex hormones or endogenous opioid function have all been proposed.^27^, ^28^, ^29^ Further research is required to explore if there is any specific mechanism in FSHD1 contributing to gender difference in pain.^30^

Pain was reported most frequently in the shoulders and lower back. These localizations are consistent with the findings of previous studies.^10^ We hypothesize that the degree of pain in these two locations may be exacerbated by the fact that the muscles in this region are amongst the weakest muscle groups in FSHD1. The abnormal posture, with forward shoulders and exaggerated lumbar lordosis as a consequence of the weakness, may be a cause of pain. Lumbosacral spine movements and kinetics are essential to normal movement; therefore, lower back pain might negatively impact on the patients' ability to stand or walk. This type of pain could be partially due to the asymmetrical, multifocal pattern of muscle involvement that in itself affects movement, generating a circle of pain, muscle atrophy, and functional impairment. Muscular involvement of the hands is less common in FSHD1^31^; therefore, it is unsurprising that this was an area where pain was less frequently reported. These findings and the possible correlation of pain with the areas of greater weakness suggest an interesting area for future study. The development of specific strategies to improve strength, flexibility, and endurance in the areas most affected by pain, could lead to an improvement in the patient's mobility as well as QoL.

FSHD1 is an inheritable muscular condition with a progressive clinical course; therefore, we would expect to see an association between chronic pain and disease duration. No correlation was seen between chronic pain and current age, age of onset or disease duration. We may speculate that the causes of pain in different age groups and in patients with different disease duration times may be attributed to different causes with inflammatory mechanisms and muscle pain probably being more important in the early stages of disease and mechanical problems associated with asymmetrical muscle atrophy and weakness being the cause for pain later in the natural history of the disease. These aspects need to be considered in future studies to achieve an optimal management of pain.

Chronic pain and the severity of it were not significantly correlated with the D4Z4 fragment size or motor function; therefore, our data did not confirm the relationship between genetic pattern, patient ambulatory state, and chronic pain. Conversely, when considering “current pain” both with the SF‐MPQ and the universal pain assessment tool, patients who had no mobility limitations had a tendency to report less pain, as previously reported^10^; but in terms of chronic pain, the frequency of pain is similar in ambulatory and nonambulatory patients. This controversial data warrant further studies to carefully assess the presence of different type of pain in FSHD1 patients.

More than 90% of the patients reported taking medication, the most common treatments were NSAIDs and opioids, used more frequently than in previous studies. Physiotherapy was used to control pain in half of the patients together with other interventions. Although physiotherapy was reported as beneficial by some patients, a small percentage reported an exacerbation of the symptoms after the treatment; whether this worsening is due to lack of access to specialist physiotherapy for neuromuscular diseases should be assessed. It is necessary to identify whether there is a subgroup of FSHD1 patients who may get worse with physiotherapy to improve the therapeutic approach to these patients. On the other hand, worsening of the pain due to disease progression may be incorrectly attributed to therapy by some patients. Our data confirm that there is no consistent management of pain in FSHD1 and that current methods are not adequately relieving pain. Evaluating the patient's response to individual treatment is warranted to develop optimal pain therapy regimens in FSHD1 patients.

We have estimated QoL in this cohort using INQoL. There are difficulties for assessing QoL in neuromuscular diseases; therefore, there are few comprehensive studies available. In general, it is reported that QoL in patients with neuromuscular diseases is low.^7^, ^14^, ^32^, ^33^ INQoL has been highly rated in terms of its conceptual and measurement model, reliability, validity, and administrative burden in neuromuscular diseases.^20^ INQoL provides an overall score along with results in different domains. It should be noted that INQoL was not designed specifically for FSHD patients and, therefore, is limited as a true measure of QoL for FSHD. Specifically, INQoL includes questions regarding muscle locking, which has no relevance to this population. In our population, the INQoL domains having the biggest impact were muscle weakness, activities, and body image. The domain least affected, apart from muscle locking, was relationships. Our study has shown that patients perceive a deterioration in QoL with the progression of the disease; the younger the age of onset and the longer the disease duration, the higher the patients' perception of disability. Moreover, the perception of lower QoL was also related to lower *Eco*RI fragment sizes. These results suggest a relationship between genetic pattern, clinical severity, and perception of QoL.

The analysis of our data shows that chronic pain has a major negative impact on the QoL in FSHD1 consistent with the literature.^13^, ^15^ Future research should focus on the identification of the features of pain with the greatest impact on QoL. This may support the development of effective treatments.

In this study, all the questionnaires were patient reported^17^ without input from clinicians. This gives information about how patients perceive pain and QoL. PROM are an extraordinarily useful tool, favored by regulatory agencies, for monitoring the impact of care on patient well‐being and Qol.^34^

There are limitations to patient‐reported data and the element of self‐selection to the registry should not be ignored. An important limitation of the current study is the lack of information on physical examination/strength measures; more severely clinically affected individuals may be more likely to experience shoulder and back pain. The patient population may represent the most engaged and active patients who may not be representative of the entire FSHD1 population; however, our data are in line with previously published studies. Furthermore, our study could be improved through the inclusion of longitudinal data, assessing changes in pain and QoL over time. Finally, one of the pain questionnaires (universal pain assessment tool) has not yet been validated; however, there was a good correlation with the well‐validated SF‐MPQ. In answering the universal pain assessment tool, it is important to clarify that patients have to recognize the pain as a result of their FSHD, and that it may be difficult to differentiate this pain from other types of pain.

In conclusion, pain is a frequent symptom in FSHD1 that negatively impacts QoL. Studies have demonstrated that pain in FSHD1 is an inherent feature of the condition that requires further investigations to understand the pathophysiology, to study the relation with disease severity and functional disability.

## Supporting information

Additional supporting information may be found in the online version of this article.

Supporting Information Table S1
