# Supplementary material for: Chronic pain has a strong impact on quality of life in facioscapulohumeral muscular dystrophy
Source: Muscle Nerve. 2017 Nov 7;57(3):380–7. doi: 10.1002/mus.25991 (PMC5836962; doi:10.1002/mus.25991)
Supplement: Supplementary file 1 — Supporting Information Table S1 [file MUS-57-380-s001.docx]

Table S1: Parts of the universal pain assessment tool used in the study.

1. **Current pain**: “Please describe the greatest level of pain you have experienced in the last seven days because of your FSHD in the following locations”:
   - 1. Shoulders.
     2. Lower back.
     3. Arms.
     4. Hands.
     5. Hips.
     6. Legs.
     7. Feet.

- Patients have to choose one of the following response in each location:
  - 1. No pain.
    2. Mild.
    3. Discomforting.
    4. Distressing.
    5. Horrible.
    6. Excruciating.

1. **Chronic pain**: “The following questions are about any persistent pain you have experienced in the last five years as a result of your FSHD. This is daily pain that can be intermittent or constant that you experience for at least 12 weeks within a year (e.g. three periods of daily pain that last for four weeks or more). Have you experienced persistent pain in the last five years?”
   - 1. Yes
     2. No
   - If yes, patients have to report all the areas they experience this pain:
     1. Shoulder joint.
     2. Arm joint.
     3. Hip.
     4. Knee joint.
     5. Foot
   - Patients have to describe the level of overall chronic pain they have experienced:
     1. Mild.
     2. Discomforting.
     3. Distressing.
     4. Horrible.
     5. Excruciating
2. **Medications**: “Have you used any over the counter or prescription medications for more than one week in the last five years to help deal with persistent pain?”
   - Participants were asked to indicate all of 14 specific pain treatments used:
     1. Paracetamol.
     2. Amitriptyline.
     3. Co-codamol (combination of paracetamol and codeine).
     4. Buprenorphine.
     5. Tramadol.
     6. Pregabalin.
     7. Gabapentin.
     8. Morphine.
     9. Nortiptyline.
     10. Codeine.
     11. Aspirin.
     12. Diazepam.
     13. Ibuprofen.
     14. Other.
3. **Non-pharmacological interventions**: “Have you used any alternative therapies/treatments for more than one week in the last five years to help deal with persistent pain?”
   - Participants were asked to indicate all of 13 specific pain therapies used:
     1. Acupuncture.
     2. Exercise.
     3. Range of motion.
     4. Counselling.
     5. Massage.
     6. TENS (transcutaneous electric nerve stimulation).
     7. Strengthening.
     8. Heat.
     9. Hydrotherapy.
     10. Chiropractic.
     11. Marijuana.
     12. Biofeedback.
     13. Other.
4. **Physiotherapy**: “The following questions discuss any physiotherapy you have received to help deal with pain you experience as a result of your FSHD. This does not include any therapy you have received as part of rehabilitation for other illness or injury.
   - “Have you received any physiotherapy to help deal with the pain you experience as a result of your FSHD?”
     1. Yes.
     2. No.
   - If yes, patients have to answer to “Did you see a reduction in the pain you experienced due to physiotherapy?”
     1. A dramatic reduction in pain.
     2. Some reduction in pain.
     3. No reduction in pain.
     4. Pain increased.
